# Directional preference in dogs: Laterality and "pull of the north"

**DOI:** 10.1371/journal.pone.0185243

**Published:** 2017-09-25

**Authors:** Jana Adámková, Jan Svoboda, Kateřina Benediktová, Sabine Martini, Petra Nováková, David Tůma, Michaela Kučerová, Michaela Divišová, Sabine Begall, Vlastimil Hart, Hynek Burda

**Affiliations:** 1 Department of Game Management and Wildlife Biology, Faculty of Forestry and Wood Sciences, Czech University of Life Sciences, Kamýcká 129, Praha 6, Czech Republic; 2 Department of General Zoology, Faculty of Biology, University of Duisburg-Essen, Essen, Germany; University of New England, Australia, AUSTRALIA

## Abstract

Laterality is a well described phenomenon in domestic dogs. It was shown that dogs, under calm Earth's magnetic field conditions, when marking their home ranges, tend to head about north- or southwards and display thus magnetic alignment. The question arises whether magnetic alignment might be affected or even compromised by laterality and vice versa. We tested the preference of dogs to choose between two dishes with snacks that were placed left and right, in different compass directions (north and east, east and south, south and west or west and north) in front of them. Some dogs were right-lateral, some left-lateral but most of them were ambilateral. There was a preference for the dish placed north compared to the one placed east of the dog ("pull of the north"). This effect was highly significant in small and medium-sized breeds but not in larger breeds, highly significant in females, in older dogs, in lateralized dogs but less significant or not significant in males, younger dogs, or ambilateral dogs. Laterality and “pull of the north” are phenomena which should be considered in diverse tasks and behavioral tests with which dogs or other animals might be confronted. The interaction and possible conflict between lateralization and "pull of the north" might be also considered as a reason for shifted magnetic alignment observed in different animal species in different contexts.

## Introduction

Laterality, i.e. the predictable, non-random preference for using one side of the body (limbs, brain hemisphere, sensory organs) spontaneously or if forced or restricted to choose between two sides has been intensively studied and is well described in humans but it seems to be also a widespread phenomenon among animals. Laterality may be inborn, imprinted or entrained and has to be taken into account in maze and behavioral two-choice animal experiments [1–5]. The laterality effect is often tested (and excluded) by counting the animals' choice for either side of a T- or Y-maze under control conditions (e.g. without a stimulus or reward) and/or under conditions where the stimulus or reward is randomly alternating between both arms of the maze. While such a behavioral test for laterality (and the exclusion of its effect) is a standard in two-choice-experiments of this kind, potential preference for a certain (magnetic) compass direction remains usually unconsidered, and this in spite of the fact that magnetic compass preference (displayed in the so-called magnetic alignment) has been documented in a wide array of animal species in diverse behavioral contexts, reviewed in [6–10]. On the other hand, however, laterality effects should be considered also in studies dealing with compass orientation and navigation [9].

Recently, we have shown that dogs, under calm Earth's magnetic field conditions, when marking their home ranges tend to head about north- or south-wards and display thus magnetic alignment [10]. In that particular study, a test for laterality was not relevant and not necessary to exclude the existence and significance of magnetic alignment. Nevertheless, we cannot exclude that laterality played a role under some circumstances and could have influenced the angularity of the response—e.g. does a "left-handed" dog turn southwards if it comes from east but northwards if it comes from the west? Laterality in dogs was examined behaviorally in more than 20 studies e.g. [11–23]. The most commonly used motor test was the so-called “Kong test”, in which the preference for a paw holding a Kong (a toy stuffed with food, KONG Company) is recorded. This laterality test is most probably not influenced by magnetoreception, cannot be masked by it but, at the same time, cannot be used to address the question whether laterality affects directional preference in the context of long-distance locomotor spatial orientation. However, there are also some other tests of lateralization—e.g. preference to approach food items placed either right or left of the dog [12] and studies of performance requiring movement in a prescribed direction [23] which can theoretically be affected or even be compromised by magnetic alignment.

## Materials and methods

No permits were required for the described study, which complied with all relevant regulations. All the dog owners were informed about the study, consented with the set-up and use of their dog(s) and were present at trials.

Altogether 25 dogs (12 M, 13 F) of 14 breeds, aged on average 5.3 (SD 3.3, 1–12) years were tested in the Czech Republic (12 dogs) and in Germany (13 dogs) (Table 1, S1 Table). The 12 dogs in the Czech Republic were tested at altogether 23 localities (distinct cities and country districts, each dog in 36 test series at each locality), the dogs in Germany were tested at altogether eight localities, each dog in 20 test series at each locality. At each locality, the dogs were tested at at least two different sites. Test series were performed at different places at each site, at different days over several months, at different times of the day, so that tests with each dog evenly covered all daytimes. In all the cases, study sites were open fields, away from communications, buildings, high voltage power lines and conspicuous landmarks. Altogether 1,088 test series were performed.

**Table 1:** Survey of the tested dogs and their factors.

breed	subject	country	size	sex	age	laterality	n test series	μ (1st locality)	μ (2nd locality)
borzoi	Hen	CZ	L	M	1	left	36 + 36	**225°**	**135°**
labrador retriever	Mon	CZ	L	M	12	ambi	36	**72°**	**x**
wirehaired pointer	Aja	CZ	L	F	4	ambi	36 + 36	**220°**	**305°**
beagle	Azi	CZ	M	M	4	right	36 + 36	**354°**	**45°**
cocker spaniel	Bar	CZ	M	F	2	left	36 + 36	**353°**	**18°**
cocker spaniel	Nel	CZ	M	F	8	ambi	36 + 36	**281°**	**278°**
foxterrier	Fre	CZ	M	F	1	left	36 + 36	**54°**	**333°**
foxterrier	Gof	CZ	M	F	1	left	36 + 36	**45°**	**62°**
foxterrier	Bes	CZ	M	F	6	right	36 + 36	**18°**	**349°**
dachshund	Ter	CZ	S	F	4	ambi	36 + 36	**270°**	**257°**
dachshund	Can	CZ	S	F	6	ambi	36 + 36	**323°**	**315°**
dachshund	Ven	CZ	S	F	9	right	36 + 36	**349°**	**333°**
border collie x münsterlander	Paa	GER	L	F	9	ambi	20	**146°**	**x**
bullterrier	Gis	GER	L	F	10	ambi	20	**135°**	**x**
flat-coated retriever	Fje	GER	L	M	2	ambi	20	**270°**	**x**
labrador retriever	Lor	GER	L	M	3	ambi	20	**180°**	**x**
labrador retriever x rottweiler	Jim	GER	L	M	3	ambi	20	**90°**	**x**
labrador retriever x rottweiler	Sam	GER	L	M	3	ambi	20	**135°**	**x**
labrador retriever	Pal	GER	L	M	5	ambi	20	**101°**	**x**
labrador retriever	Lot	GER	L	F	7	left	20	**54°**	**x**
jack russel terrier	Jac	GER	M	M	12	left	20	**31°**	**x**
chihuahua	Att	GER	S	M	3	right	20	**72°**	**x**
chihuahua	Ger	GER	S	M	4	right	20	**0°**	**x**
chihuahua	Hen	GER	S	M	5	ambi	20	**0°**	**x**
yorkshire terrier	Bri	GER	S	F	10	ambi	20	**31°**	**x**

Size: L = large, M = medium, S = small, Sex: F = female, M = male, Age is given in years, laterality: ambi-, left- or right-lateral, n = number of test series (in each test series 4 trials were performed), μ = mean vector in ° at the first locality and, if tested, at the second (distant) locality; at each of those distant localities 36 test series were performed., x = the dog was not tested at the second locality. The column “subject” refers to the first three letters of the respective dog’s name. See the supporting information (S1 Table) for further detail.

**Fig 1:**
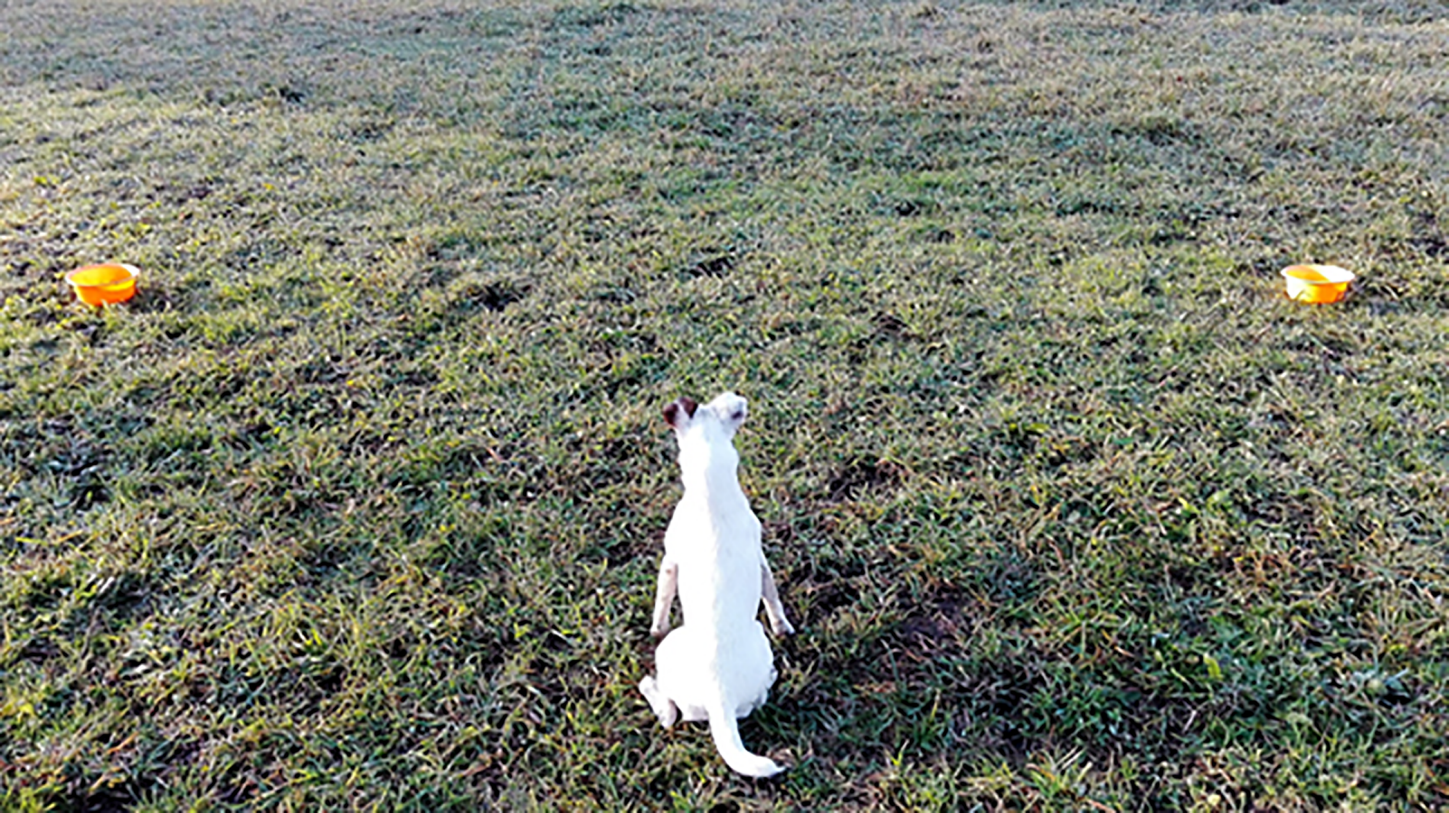
Photo illustrating the study setup.

Each test series involved 4 trials where the dog should make a choice between two identical dishes placed in front of it, at a distance of 2–6 m, according to the size of the dog, so that it could not look into the dishes from its starting point. The dishes were placed in an angle plus and minus 45° from the starting point, so that one dish was e.g. eastwards of the dog and the second one was placed southwards. Each test series involved four trials (test combinations): north versus east, east versus south, south versus west, and west versus north. The sequence of the tests was changed randomly. Both dishes contained identical dog snack. The dog could not see the preparation of the test, i.e. the placement of the dishes. The dog was brought to the starting point and waited to get permission to go to the dish (of its choice). Two experimenters were involved in this test. The first experimenter was setting up the test, while the dog owner (who was uninformed about the actual directions of the dishes and gave the voice command) was either standing behind the dog, and had no eye contact with it, in the Czech Republic (Fig 1), or had dark sun glasses and stood on the opposing side of the dishes, facing the dog, in Germany.

All the dog owners were informed about the study, consented with the set-up and use of their dog(s) and were present at trials. The owners in the Czech Republic were coauthors of this study, their colleagues, and friends; the dog owners in Germany were approached via dog-training clubs.

Only the direction of the first choice was recorded. The dog was allowed to visit and take the snack also from the other dish. Apart from the chosen first direction in a given combination, the sequence of tested combinations within the trial, the dog's and owner's identities, locality, place, date, daytime, weather (sunny, cloudy, overcast, rainy), wind strength and direction were recorded. (However, no tests were performed on windy days.)

From the recorded choices, preferences for either left or right turn were calculated for all test combinations (N-E, E-S, S-W, W-N) within each trial, and the sum for all trials for each dog. Index of laterality was then calculated for each dog according to the formula (R–L) / (R + L) x 100, where R and L is the preference for using the right and left side, respectively [11]. Significance of lateralization was tested by chi-square test.

Independently, mean directional compass preference based on the frequency of first choices at a given locality in all pooled trials (at different day times, different days, different places within the locality) was calculated for each dog using circular statistics with Oriana 4.02 (Kovach Computing). Grand mean vectors were then calculated on the base of those mean dog/locality vectors for all the dogs, and subgroups with respect to laterality, breed, body size, sex, and age.

## Results

### Laterality

In dish-choice trials, altogether six dogs were identified as left-sided, five dogs as right-sided and 14 dogs were ambilateral based on the choices the respective dogs made in approaching one of the dishes placed left and right in front of them. There was no clear effect of breed and sex on laterality.

### Compass preference

**Fig 2:**
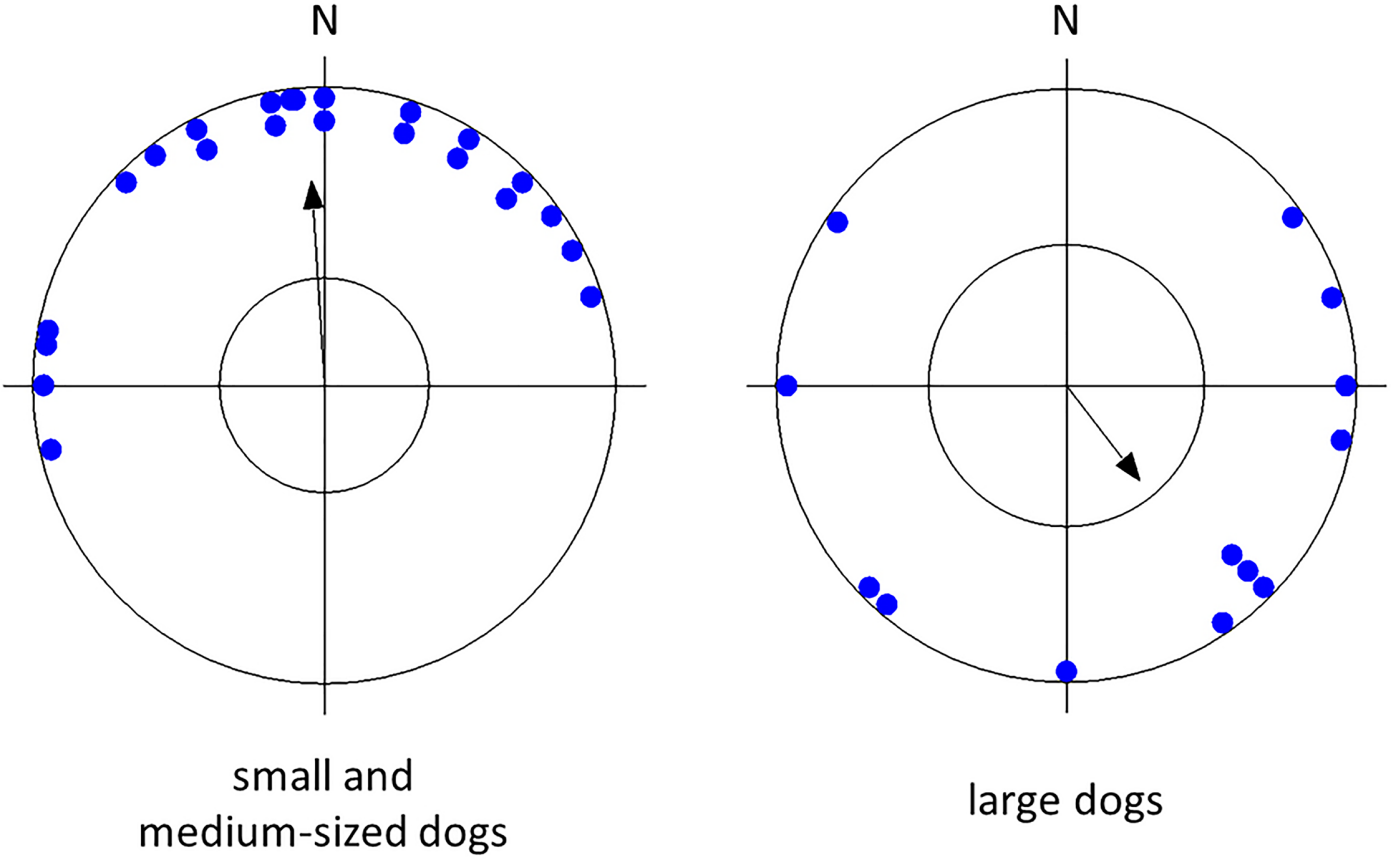
Mean preference for compass direction of a dish with snacks of the first choice. **Angular means over dogs of small, medium-sized, and large breeds.** The arrow indicates the grand mean axial vector (μ) calculated over all angular means. The length of the mean vector (r) provides a measure of the degree of clustering in the distribution of the mean vectors. The inner circle marks the 0.05 level of significance border of the Rayleigh test. See Table 2 for statistics.

**Fig 3:**
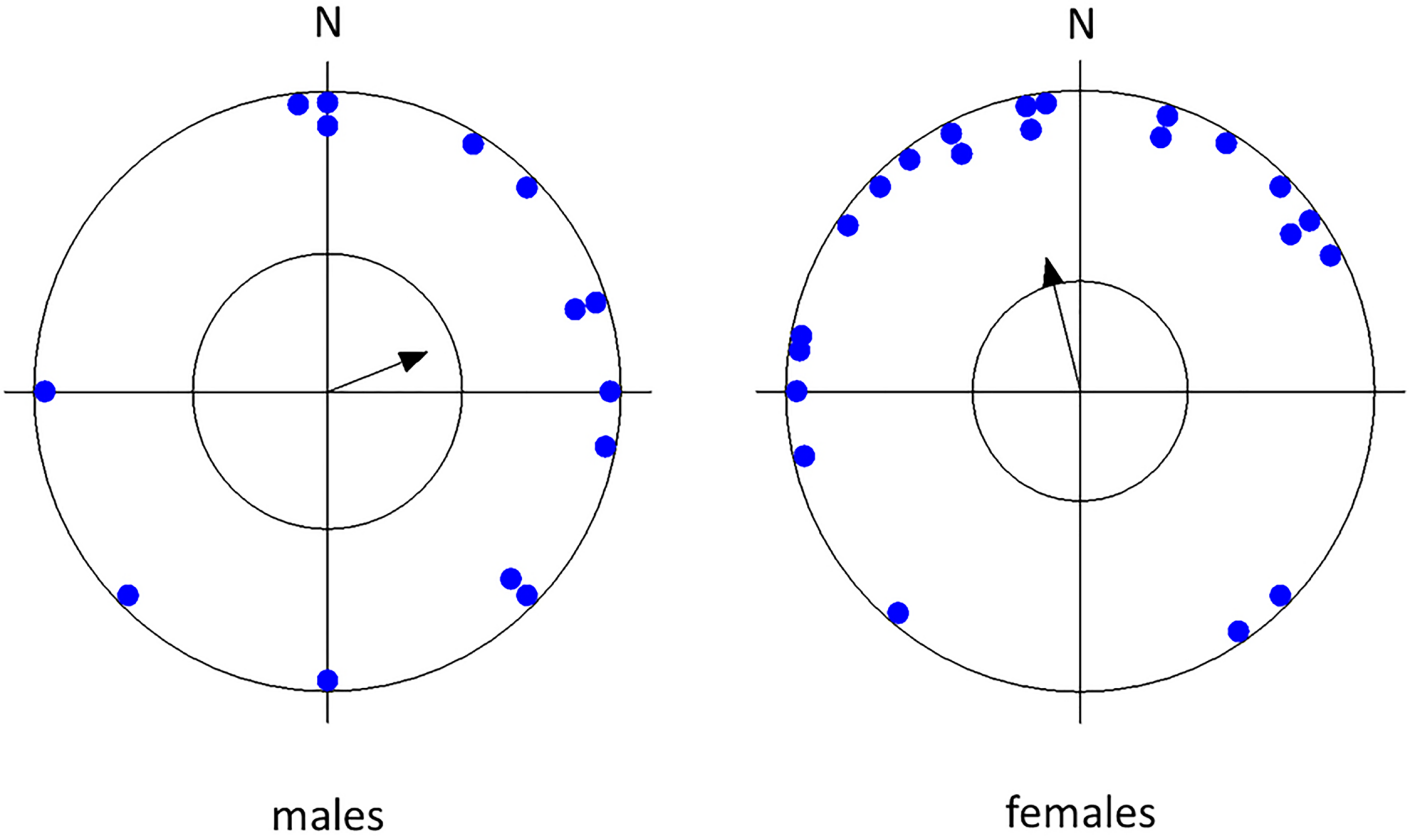
Mean preference for compass direction of a dish with snacks of the first choice. **Angular means over males and females separately.** The arrow indicates the grand mean axial vector (μ) calculated over all angular means. The length of the mean vector (r) provides a measure of the degree of clustering in the distribution of the mean vectors. The inner circle marks the 0.05 level of significance border of the Rayleigh test. See Table 2 for statistics.

**Fig 4:**
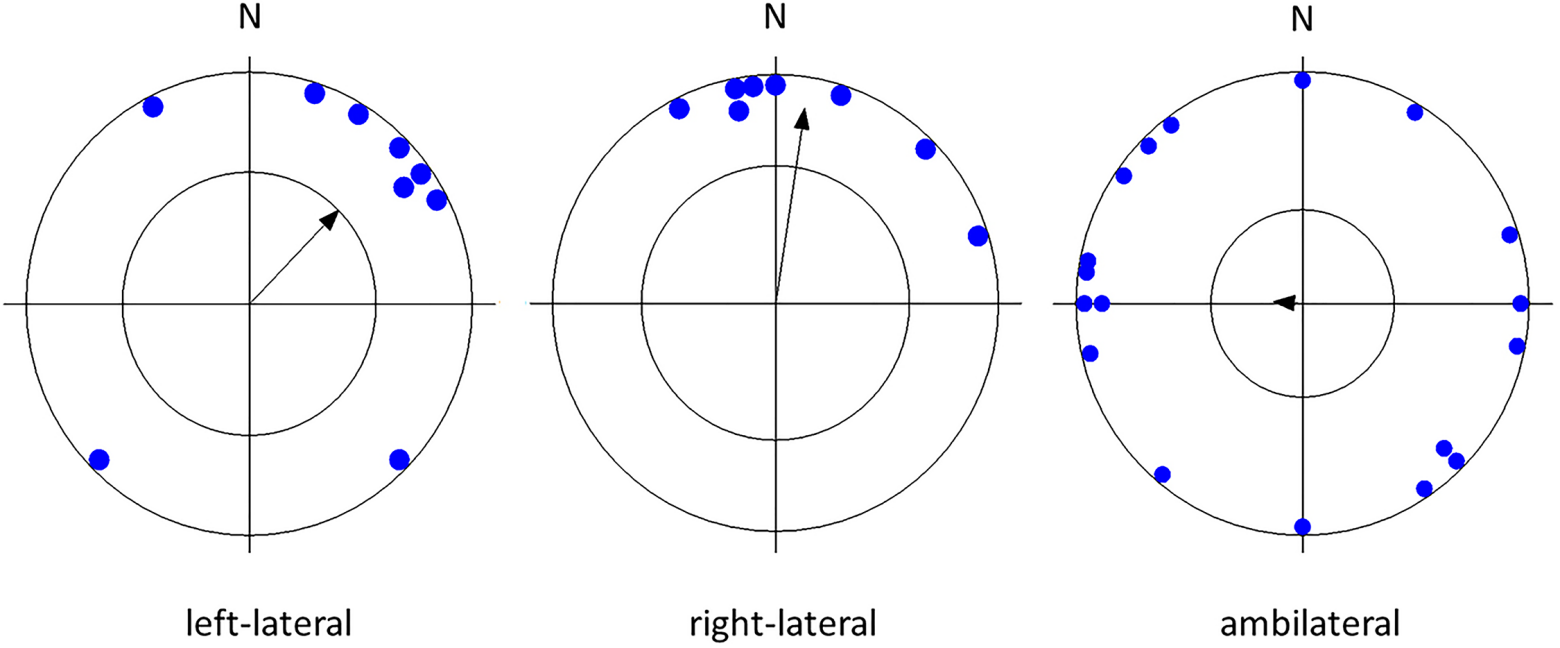
Mean preference for compass direction of a dish with snacks of the first choice. **Angular means over dogs of different lateralization types.** The arrow indicates the grand mean axial vector (μ) calculated over all angular means. The length of the mean vector (r) provides a measure of the degree of clustering in the distribution of the mean vectors. The inner circle marks the 0.05 level of significance border of the Rayleigh test. See Table 2 for statistics.

**Fig 5:**
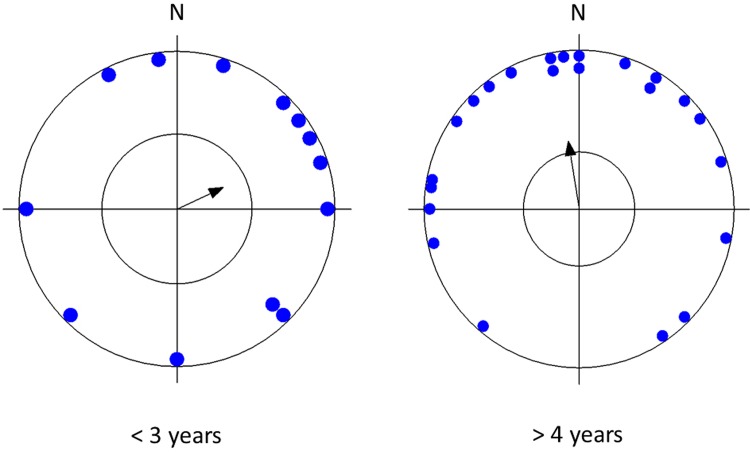
Mean preference for compass direction of a dish with snacks of the first choice. **Angular means over dogs of different age categories.** The arrow indicates the grand mean axial vector (μ) calculated over all angular means. The length of the mean vector (r) provides a measure of the degree of clustering in the distribution of the mean vectors. The inner circle marks the 0.05 level of significance border of the Rayleigh test. See Table 2 for statistics.

Testing the circular distribution of mean vectors of all dogs, as well as of dogs of a particular lateralization, body size, sex, and age revealed that there was an apparent preference for the north ("pull of the north") which was highly significant in small and medium-sized breeds but not in larger breeds, highly significant in females, in older dogs, in lateralized dogs but less significant or not significant in males, younger dogs, or ambilateral dogs (Table 2, Figs 2–5).

**Table 2:** Circular statistics for frequencies of choices of a dish placed in north or east or south or west in front of a dog in dual choice experiments where the dog chose between north or east, east or south, south or west, west or north.

Variable	all dogs	males	females	small- and medium-sized	large- sized	ambi- lateral	left- lateral	right- lateral	< 3 years	> 4 years
**Number of observations**	36	14	22	23	13	18	10	8	13	23
**Mean vector (μ)**	11°	68°	345°	356°	142°	274°	37°	9°	65°	350°
**Length of mean vector (r)**	0.328	0.362	0.457	0.686	0.405	0.128	0.578	0.862	0.322	0.432
**Circular standard deviation**	86°	82°	72°	50°	77°	116°	60°	31°	86°	74°
**Rayleigh test (Z)**	3.868	1.832	4.593	10.821	2.135	0.294	3.338	5.939	1.351	4.295
**Rayleigh test (p)**	0.017	0.161	0.009	4.8 x 10^−6^	0.118	0.750	0.031	6.79x10^-4^	0.264	0.012

Each compass direction was offered with the same frequency. The number of observations refers to the number of dogs and localities. Mean vectors in this table represent thus grand mean vectors.

Testing the frequencies of compass preferences combining always two neighboring test combinations centered at one compass direction (e.g. triad W-N-E combined results of tests W-N and N-E, and frequency of choices 25–50–25% would be expected if there were no preferences and no laterality) revealed statistically significant difference from random distribution in tests involving North and East (W-N-E, N-E-S, chi-square p = 0.0001 and 0.0022, respectively) but not in both other combinations (E-S-W, p = 0.5011; S-W-N, p = 0.1446).

## Discussion

We tested the preference of dogs to choose between two dishes with snacks that were placed left and right, in different compass directions (north and east, east and south, south and west or west and north) in front of them. Some dogs were right-lateral, some left-lateral but most of them were ambilateral. There was a preference for the dish placed north compared to the one placed east of the dog ("pull of the north"). This effect was significant in small and medium-sized breeds but not in larger breeds, significant in females, in older dogs, in lateralized dogs but not significant in males, younger dogs, or ambilateral dogs. None of the extrinsic factors which might have influenced the directional choice, but the Earth's magnetic field, was stable in space and time. All other factors (possible hints from the owners, landmarks, sun position, weather, homing direction) were stochastically changing and could not have systematically influenced the observed "pull of the north".

### His master's voice?

The test was performed by several different dog owners. We do not see any possibility how the dogs might have been systematically (i.e. in one common direction and only in a compass combination involving north and east) influenced in their decision by their owners. The dogs had no eye contact with their owners. In fact, every choice was rewarded, and the dogs were allowed to take also the reward from the second dish, so that effective learning, e.g. on some landmark in the surroundings, was not possible. Besides that, the next trial occurred at another place, or site or even at another locality, at another day, another day-time, and the sequence of tested compass direction pairs changed.

### Effect of sun?

"Pull of the north" could be theoretically explained as avoidance of blinding sun. This explanation is rather anthropomorphic and does not consider the fact that dogs are lower than human and that the dishes were placed on the ground. Moreover, this argument would be valid only in the choice test "north x east" on sunny mornings in spring and autumn when sun is low. Analogously, however, preference for the north would be expected also in the choice test "north x west" in the evening on sunny days. Furthermore, dogs would be expected to avoid south during midday on sunny days. Larger (higher) dogs are expected to be more prone to blinding than smaller (lower) dogs. Apart from the fact that actually there was never a choice test "north x south" or "east x west" where sun avoidance could be actually realized, none of these possible explanations for the results was supported. Circular analysis of the tests performed in "lateral" dogs in the morning (grand mean vector μ = 42°, r = 0.510, SD = 66, Rayleigh test p = 0.041, N = 12) and in the afternoon (grand mean vector μ = 22°, r = 0.650, SD = 53, Rayleigh test p = 0.0006, N = 16) revealed no differences (99% confidence interval for μ a.m. 343°-100°, for p.m. 347°-57°). Also "ambilateral" dogs displayed no differences between morning and afternoon choices (morning: μ = 286°, p = 0161, N = 12; afternoon = 269°, p = 0.488, N = 15). There were no significant differences between whatever combination of directions (N x W, N x E, S x E, S x W) tested in the morning and in the afternoon, respectively.

Note also that in Central Europe, Czech Rep. and Germany, where the experiments were done, the sun hours make on average only about 35% of the light hours—so, if the experiments are randomly and evenly distributed throughout the year and daytimes, the probability of being blinded by the sun (if one lifts the head or the sun is low and one looks into it), is 35%. The probability that one actually has problems to recognize where on the sky the sun stands is 65%. Note also that according to the hitherto knowledge dogs do not perceive polarized light.

### Other possible effects?

The Earth's magnetic field parameters in both countries are comparable. The declination in Essen (Germany) is 1.5°, the declination in Prague (Czech Rep.) is 3.5°, and the difference is thus only 2°. The dogs had to decide, however, for one of the two directions which were 90° apart. Most importantly, directions in the field in both countries were measured by means of a compass which shows *always* a direction to the magnetic pole, i.e. not by means of a geographical map which would show direction to the geographic pole.

Although in Germany relatively more larger dogs were represented in the sample than in the Czech Republic, there was no apparent country bias if smaller versus larger, lateral versus ambilateral, etc. dogs in the two countries, were compared. In spite of the large dataset, the subgroups (breeds, age, sex, lateralization) were still rather small and heterogeneous and did not enable us to reliably test which of those facts was most decisive. We have also no idea why larger breeds "failed" in the compass test. It should be noticed that this category was composed mainly of (labrador) retrievers but it would be preliminary to claim that this breed is ambilateral and/or "magnet-blind". The fact that older dogs oriented more towards the north than younger dogs might correlate with their experience and established orientation strategies.

We have purposely performed the experiments outdoors because the magnetic field in buildings might be disturbed and because dogs tested indoors might orient with respect to geometry of rooms. Since it cannot be excluded that dogs might orient with respect to landmarks also outdoors, care was taken when selecting the testing place that its surroundings were free of any landmarks within the radius of at least 30 m, a condition which could not be complied with indoors.

### Conclusions and prospects

The here presented two-dish choice test widens the range of tests reviewed in [12, 24] suitable for assessment of lateralization in dogs. At the same time it shows that laterality plays a role not only in intrinsic kinesthetic reactions but also in directional goal-oriented decisions. Moreover there is an apparent "pull of the north", which is particularly strong in the test combination "north—east". Laterality and "pull of the north" are thus phenomena which should be considered in diverse tasks (and behavioral tests) with which dogs or other animals might be confronted. On the other hand, the phenomenon of laterality should be likewise considered in studies of spatial orientation and navigation. The interaction and possible conflict between lateralization and "pull of the north" might be also considered as a reason for shifted magnetic alignment observed in different animal species in different contexts [9]. It might prove to be significant for understanding the putative (and thus far unknown) mechanism of magnetoreception that the field observations and laboratory experiments in diverse animal species consistently show a shift of directional preference from north or south to the east after a treatment with a strong magnetic pulse e.g. [25–26]. Moreover, roe deer were found to prefer to escape northwards but to avoid escaping eastwards [27]. These (and further own unpublished) observations indicate some sensory interaction between perceiving north and east directions.

## Supporting information

S1 Table Information on tested dogs, particulars of trials and their results. Age is given in years, the choice for a dish in the respective combination in compass degrees.
(XLSX)

## References

[1] Rogers LJ. Laterality in animals. Internat J Comp Psychol. 1989;3:5–25.

[2] Rogers LJ. Lateralization in vertebrates: its early evolution, general pattern, and development. Adv Study Behav. 2002;31:107–161.

[3] Rogers LJ, Vallortigara G, Andrew R.J. Divided brains. The Biology and Behaviour of Brain Asymmetries. 2013;Cambridge University Press, New York.

[4] Schaafsma SM, Riedstra BJ, Pfannkuche KA, Bouma A, Groothuis TG. Epigenesis of behavioural lateralization in humans and other animals. Phil Trans Roy Soc B. 2009;364:915–927. 19064352 10.1098/rstb.2008.0244 PMC2666084

[5] Warren JM. Handedness and laterality in humans and other animals. Physiol. 1980;8:351–359.

[6] Begall S, Malkemper EP, Červený J, Němec P, Burda H. Magnetic alignment in mammals and other animals. Mammal Biol. 2013;78:10–20.

[7] Begall S, Malkemper EP, Burda H. Magnetoreception in mammals. Adv Study Behav. 2014;46:45–88.

[8] Wiltschko R, Wiltschko W. Magnetic orientation in animals 1995; Springer, Berlin.

[9] Malkemper EP, Painter MS, Landler L. Shifted magnetic alignment in vertebrates: Evidence for neural lateralization? J Theor Biol. 2016;399:141–147. doi: 10.1016/j.jtbi.2016.03.040 27059891 10.1016/j.jtbi.2016.03.040

[10] Hart V, Nováková P, Begall S, Malkemper EP, Hanzal V, Ježek M, et al Dogs are sensitive to small variations of the Earth's magnetic field. Front Zool. 2013;10:80 doi: 10.1186/1742-9994-10-80 24370002 10.1186/1742-9994-10-80 PMC3882779

[11] Batt L, Batt M, McGreevy P. Two tests for motor laterality in dogs. J Vet Behav. 2007;2:47–51.

[12] Berta C. Lateralized behavior in domesticated dogs. ESSAI. 2010;8:9.

[13] Branson NJ, Rogers LJ. Relationship between paw preference strength and noise phobia in *Canis familiaris*. J Comp Psychol. 2006;120:176–183. doi: 10.1037/0735-7036.120.3.176 16893254 10.1037/0735-7036.120.3.176

[14] Nagasawa M, Kawai E, Mogi K, Kikusui T. Dogs show left facial lateralization upon reunion with their owners. Behav Proc. 2013;98:112–116. 10.1016/j.beproc.2013.05.012 23727034

[15] Plueckham TC, Schneider LA, Delfabbro PH. Assessing lateralization in domestic dogs: Performance by *Canis familiaris* on the Kong test. J Vet Behav. 2016;15:25–30.

[16] Schneider LA, Delfabbro PH, Burns NR. Temperament and lateralization in the domestic dog (*Canis familiaris*). J Vet Behav. 2013;8:124–134.

[17] Siniscalchi M, Sasso R, Pepe AM, Vallortigara G, Quaranta A. Dogs turn left to emotional stimuli. Behav Brain Res. 2010; 208:516–521. doi: 10.1016/j.bbr.2009.12.042 20060016 10.1016/j.bbr.2009.12.042

[18] Siniscalchi M, Quaranta A, Rogers LJ. Hemispheric specialization in dogs for processing different acoustic stimuli. PLoS ONE. 2008; 3:e3349 doi: 10.1371/journal.pone.0003349 18843371 10.1371/journal.pone.0003349 PMC2553184

[19] Siniscalchi M; Pergola G, Quaranta A. Detour behaviour in attack-trained dogs: Left-turners perform better than right-turners. Laterality. 2013; 18:282–293. doi: 10.1080/1357650X.2012.662234 22713109 10.1080/1357650X.2012.662234

[20] Siniscalchi M.; d’Ingeo S.; Fornelli S.; Quaranta A. Relationship between visuospatial attention and paw preference in dogs. Sci Rep 2016, 6 10.1038/srep31682 PMC4992877 27545695

[21] Siniscalchi M, d’Ingeo S, Quaranta A. The dog nose “KNOWS” fear: Asymmetric nostril use during sniffing at canine and human emotional stimuli. Behav Brain Res. 2016; 304:34–41. doi: 10.1016/j.bbr.2016.02.011 26876141 10.1016/j.bbr.2016.02.011

[22] Tomkins LM, Thomson PC, McGreevy PD. First-stepping Test as a measure of motor laterality in dogs (*Canis familiaris*). J Vet Behav. 2010; 5:247–255.

[23] Tomkins LM, Williams KA, Thomson PC, McGreevy PD. Lateralization in the domestic dog (*Canis familiaris*): Relationships between structural, motor, and sensory laterality. J Vet Behav. 2012;7:70–79.

[24] Rogers L, Vallortigara G (Eds). Lateralized Brain Functions. Methods in Human and Non-Human Species. 2017; Springer, New York.

[25] Wiltschko R, Wiltschko W. Magnetic orientation in animals 1995; Springer, Heidelberg.

[26] Marhold S, Burda H., Kreilos I, Wiltschko W. Magnetic orientation in common mole-rats from Zambia. In: Orientation and Navigation: Birds, Humans and Other Animals. 1997; Paper No 5. Royal Institute of Navigation, Oxford.

[27] Obleser P, Hart V, Malkemper EP, Begall S, Holá M, Painter MS, et al Compass-controlled escape behavior in roe deer. Behav Ecol Sociobiol. 2016;70:1345–1355.

